# Bursting neurons in the hippocampal formation convey information about LFP features

**DOI:** 10.1186/1471-2202-15-S1-P89

**Published:** 2014-07-21

**Authors:** Maria Constantinou, Daniel H Elijah, Daniel Squirrell, Inés Samengo, John Gigg, Marcelo A Montemurro

**Affiliations:** 1 Faculty of Life Sciences, University of Manchester, Manchester, M13 9PT, UK; 2 Centro Atómico Bariloche and Instituto Balseiro, San Carlos de Bariloche, 8400, Argentina

## 

Local field potentials (LFPs) are thought to reflect the combined activity of neuronal populations projecting onto a local area. Accumulating evidence suggests that LFPs can contribute to neural information encoding by providing a reference signal that can boost the information conveyed by spikes [1,2]. Although some mechanisms have been proposed to explain how this extra information might be made available to downstream neurons [3,4], they are yet to be tested against experimental data. In this study, we used both modelling of hippocampal pyramidal neurons and *in-vivo* data to investigate how neurons in the hippocampal formation can use bursting to convey information about features of LFPs. Intrinsic bursts produced by pyramidal neurons provide a graded signal determined by the burst spike count. Fitting a dual-compartment model of a pyramidal cell to realistic burst firing [3,5,6] shows that bursts with spike counts of 1 (tonic firing), 2 and 3 or more are triggered with a differential locking with respect to features of the reference LFPs. This locking provides an explicit mechanism for bursts of different spike count to transmit information contained in LFPs to downstream neurons. We tested the model predictions by analysing recordings of spontaneous LFPs and spiking activity from areas CA1 and subiculum of urethane-anaesthetised rats. Statistical analyses and information theory were used to quantify the encoding capacity of bursts to discriminate basic features of LFPs, such as phase, slope, instantaneous voltage and energy. In agreement with model predictions, features such as phase and slope were found to be the most reliable single features correlated to burst size. We also applied multiple discriminant analysis to characterise the set of optimal LFP features that were maximally discriminated by different bursts. We found that burst spike count conveyed significantly more information about these optimal LFP features compared to simple LFP parameters. Our results provide the first *in-vivo* evidence that neurons can use bursts to convert information about LFP features into a spike-count code that is suitable to be decoded by postsynaptic neurons in distant neural networks.
